# Generation of a host cell line containing a MAR‐rich landing pad for site‐specific integration and expression of transgenes

**DOI:** 10.1002/btpr.3254

**Published:** 2022-04-25

**Authors:** Claudia Oliviero, Steffen C. Hinz, Jan P. Bogen, Henri Kornmann, Björn Hock, Harald Kolmar, Gerrit Hagens

**Affiliations:** ^1^ Institute of Life Technology Haute Ecole d'Ingénierie HES‐SO Valais Wallis Sion Switzerland; ^2^ Institute for Organic Chemistry and Biochemistry Technical University of Darmstadt Darmstadt Germany; ^3^ Ferring Biologics Innovation Center Epalinges Switzerland; ^4^ SwissThera SA Epalinges Switzerland

**Keywords:** antibody, CHO cells, protein expression, recombinant protein, site‐specific integration

## Abstract

In recent years, targeted gene integration (TI) has been introduced as a strategy for the generation of recombinant mammalian cell lines for the production of biotherapeutics. Besides reducing the immense heterogeneity within a pool of recombinant transfectants, TI also aims at shortening the duration of the current cell line development process. Here we describe the generation of a host cell line carrying Matrix‐Attachment Region (MAR)‐rich landing pads (LPs), which allow for the simultaneous and site‐specific integration of multiple genes of interest (GOIs). We show that several copies of each chicken lysozyme 5'MAR‐based LP containing either BxB1 wild type or mutated recombination sites, integrated at one random chromosomal locus of the host cell genome. We further demonstrate that these LP‐containing host cell lines can be used for the site‐specific integration of several GOIs and thus, generation of transgene‐expressing stable recombinant clones. Transgene expression was shown by site‐specific integration of heavy and light chain genes coding for a monospecific antibody (msAb) as well as for a bi‐specific antibody (bsAb). The genetic stability of the herein described LP‐based recombinant clones expressing msAb or bsAb was demonstrated by cultivating the recombinant clones and measuring antibody titers over 85 generations. We conclude that the host cell containing multiple copies of MAR‐rich landing pads can be successfully used for stable expression of one or several GOIs.

## 1 INTRODUCTION

Since insulin entered the commercial market in 1982, a plethora of different therapeutic proteins such as hormones, coagulation factors, cytokines, fusion proteins as well as antibodies have been used successfully to treat diseases. Especially antibodies have gained huge interest in the past decades, becoming the best‐selling drugs in the pharmaceutical industry, reaching a market value of about US$157 billion in 2020.^1^ Complex molecules are commonly produced in mammalian cells, of which the Chinese hamster ovary cells (CHO cells) are the current workhorse for recombinant protein expression using mammalian cells.^2^, ^3^ In 2017, the majority of the antibodies with the highest market share was produced in CHO cells.^4^

Due to the continuous expansion of the commercial market for therapeutic proteins, there is a growing interest in the development of production systems with improved efficiency in terms of productivity, product quality, and high genetic stability.^5^ The classical pathway for stable cell line generation relies on random integration of the gene of interest (GOI) into the host cell genome. After having generated the genetic construct containing the transgene of interest and having transfected the cells, a stable polyclonal pool is generated by applying selective pressure to distinguish between cells that contain the GOI and cells that do not.^6^, ^7^, ^8^ The clonal selection step, involving repeated isolation, screening and characterization of single cell clones, can be considered the principal time limiting factor during the whole cell line development process.^8^, ^9^, ^10^ Therefore, many efforts have been made to optimize and shorten this process. Recent approaches are based on the use of epigenetic regulatory elements and/or the development of powerful genetic tools to improve the integration and expression of GOIs into the host genome.^11^, ^12^

Epigenetic regulatory elements have been described as DNA sequences that are able to modulate the chromatin state, thereby affecting gene expression. The major representatives of this category of such regulatory elements are locus control regions (LCRs), matrix attachment regions (MARs), insulators, ubiquitously acting chromatin opening elements (UCOEs), and stabilizing and anti‐repressor elements (STARs).^11^, ^13^, ^14^, ^15^, ^16^, ^17^, ^18^ Out of them, the MARs have been extensively studied and are often integrated as cis‐acting elements into expression vectors.^19^ MAR sequences are DNA regions that can link the nuclear matrix and are characterized by an AT‐rich core surrounded by two flanking regions with binding sites for various factors (insulators, transcription factors, nuclear matrix protein, etc.).^20^ For these reasons, MAR elements delimit topological domains, called chromatin loops, in which genes are protected from silencing and actively expressed.^13^, ^20^, ^21^ These features allow MAR sequences to sustain high and stable transgene expression when added into expression vectors.^13^ In addition, it has been shown that MARs help stable transgene integration by promoting the formation of plasmid concatemers and their integration into the cell genome by synthesis‐dependent microhomology‐mediated end joining (SD‐MMEJ). In this way, MAR elements promote both the number of cells integrating the transgene and the number of transgenes stably integrated per cell.^11^, ^22^ The application of these epigenetic elements helped to develop stable high‐producing recombinant cell lines for biopharmaceutical industry.^11^ Moreover, recent progress in genome engineering technologies increased the interest in the scientific community for the development of targeted integration (TI) techniques that allow the integration of a GOI into a specific site of the host genome. These techniques are either based on the use of site‐specific recombinases (integrases such as BxB1, Flp, PhiC31, and CRE) or nucleases (such as zinc finger nucleases, TALE nucleases, or the CRISPR–Cas system).^23^, ^24^, ^25^, ^26^, ^27^ Nucleases help the integration of transgenes into a specific genomic locus by inducing DNA double‐strand breaks and promoting the self‐repair mechanism of the cells.^28^ Known drawbacks are the challenging enzyme engineering, off‐target effects and long‐term stability after integration of the GOI.^29^, ^30^, ^31^ Site‐specific integrases recognize and bind defined recombination sites and catalyze DNA breaks, strand inversion and re‐ligation. To exploit this mechanism for transgene integration, a host cell line harboring a specific recombination site in the host cell genome (referred to as landing pad) needs to be generated. Subsequently, the corresponding integrase can be used to integrate the transgene into the landing pad. Based on the configuration of the recombination site in the host genome, it is possible to integrate the entire vector carrying the transgene or a specific cassette via recombinase‐mediated cassette exchange (RMCE).^32^, ^33^ Integrase‐based TI resulted in high stability, showed low off‐target effects and low limitation in terms of length of DNA payload.^34^, ^35^ In recent years, numerous landing pad harboring cell lines have been developed utilizing different combinations of recombination sites and integrases. Most of the developed systems have been examined by integrating msAb sequences into the landing pad to generate a production platform.^34^, ^36^, ^37^ However, these TI‐generated cell lines resulted in cell‐specific productivities ranging between 1 and 20 pg/cell/day, hence not meeting the criteria for large industrial scale productions for which stable cell lines, generated by random integration, reaching commonly productivities well above 20 pg/cell/day.^25^, ^38^, ^39^, ^40^, ^41^

To increase productivity of TI‐ based cell lines, the commonly used strategies are the inclusion of the landing pad itself into genomic regions, which promote a high and stable transcription rate (referred to as “genomic hot‐spot”) and the use of multi‐copy expression vectors for transgene integration (donor vector) to increase GOIs copies that can be integrated into the host cell.^7^, ^12^, ^36^, ^38^, ^41^, ^42^ However, the process of hot‐spot screening remains laborious and time‐consuming, and the use of large donor vectors carrying multiple copies of the GOI reduces the efficiency of integration into the LP.^41^, ^43^

In this work, we describe the generation of a host cell line containing several copies of MAR‐rich landing pads for the site‐specific integration of GOIs. First, we generated two different landing pad vectors containing the chicken lysozyme 5′ MAR, two different orthogonal recombination sites for the serine integrase BxB1 (AttB wild type, and AttB with GA mutation) and two different reporter genes (EGFP and DsRed). The MAR sequence associated with the LPs not only helps the integration of the LP itself into the host genome but also protects the LP from being silenced and facilitates transgene expression. After clonal selection, these newly generated host cell lines have been used to integrate GOIs into the landing pads. The use of two different landing pads enables the integration of two donor vectors at two independent genomic sites and the monitoring of the GOIs genomic integration. Site‐specific integration of the GOIs could be followed by monitoring the reduction of fluorescence intensity of the reporter genes. To our knowledge, this is the first reported system harboring multiple copies of MAR‐rich landing pads, which can be used for TI and independent expression of genes of interest. The system has been tested for the production of monospecific and bispecific antibodies, demonstrating the feasibility of targeting the two landing pads for the simultaneous expression of different genes.

## 2 MATERIALS AND METHODS

### 2.1 Plasmid construction

Landing pad vectors used in this study were derived from a modified pD603 vector (ATUM) containing a MAR sequence, the AttB site for BxB1 recombination and a reporter gene (EGFP or DsRed, respectively).^35^

A multiple cloning site (MCS) containing SapI‐BamHI‐BglII‐EcoRI‐HindIII‐XbaI‐SapI sites has been integrated into the pD603 vector to generate pD603_MCS. The MCS has been designed as single‐strand DNA oligos and, after annealing in a double strand oligo, it was cloned into pD603 linear vector. MAR sequence (5′ chicken lysozyme MAR) and DNA region containing AttB sites and reporter gene have been synthesized into pUC18 vectors (GenScript) and subcloned into pD603_MCS using EcoRV/AflII and BamHI/HindIII restriction sites, respectively. In addition, landing pad vectors containing AttB sites and reporter genes without MAR have been generated accordingly. Donor vectors containing AttP recombination sites for BxB1 and light/heavy chains were generated from pD607 and pD609 (ATUM). These vectors have been modified by adding an MCS as previously described, removing the SV40 promoter and subcloning the AttP sites upstream of the resistance gene using HindIII and SmaI restriction sites.

Genetic constructs encoding for the light/heavy chain of a human monoclonal antibody (msAb‐Fer, Ferring) have been synthesized by Genscript into pUC19 vectors and then subcloned into pD609_MCS and pD607_MCS, respectively, using BamHI and HindIII restriction sites. For the generation of donor vectors for bispecific antibody integration (bsAb‐Fer, Ferring), common light chain, knob heavy chain, and hole heavy chain genes were optimized for the CHO codon usage with GenSmart™ Codon Optimization from GenScript. Sequences were synthesized into an empty pUC18 vector (GenScript) and cloned into pD607_MCS and pD609_MCS using XbaI and SapI restriction sites. The common light chain sequence was cloned into pD609_MCS and knob and hole heavy chain were cloned into pD607_MCS.

The BxB1 expression vector pCAG–NLS–HA– BxB1 was purchased from Addgene (#51271). All vectors and sequences used in this study are listed in Table S6 (Supporting Information). All restriction enzymes were purchased from New England Biolabs and restriction reactions were carried out according to supplier's information.

### 2.2 Cell culture

Suspension‐adapted CHO‐S cells (kind gift from HES‐SO Valais Wallis) were maintained in CD CHO medium (catalogue number 10743029, Gibco) supplemented with 8 mM l‐glutamine_._ Routine cultures were inoculated at a cell concentration of 1 × 10^5^ cells/mL in 125 mL Erlenmeyer shake flasks in a working volume of 20 mL and cultivated at 37 °C, 10% CO_2,_ 120 rpm (25 mm shaking diameter) and 85% relative humidity. Cells were passed every 3–4 days. Cell viability and cell density were assessed using ViCell Blue counter (Beckmann Coulter).

### 2.3 Host cell line generation

For the generation of the host cell lines, LP_EGFP and LP_DsRed were linearized using AflII/BstZ17I and ApaLI/BstZ17I restriction enzymes, respectively. Approximately 2 × 10^5^ CHO‐S cells were transfected with 2 μg of LP_DsRed using a Neon microporator (1130 V, 20 ms, 3 pulses). After transfection, the cells were transferred in 12‐well plates in 1 ml of CD CHO + 8 mM of l‐glutamine and incubated at 37 °C, 10% CO_2_, 120 rpm (25 mm shaking diameter) and 85% relative humidity. Cells were transfected again as described above after 21 h after the first transfection and selected with G418 (added after 48 h from second transfection) at a concentration of 700 μg/mL for 2 weeks. After selection, the stable pool expressing DsRed was additionally transfected with 4 μg of LP _EGFP as previously described. 48 h after transfection, cells were subjected again to antibiotic selection for 2 weeks. A stable pool containing both LP_EGFP and LP_DsRed was transferred on semi‐solid medium for single cell‐derived colony picking. The same protocol described above was repeated using LP vectors that do not contain MAR sequences. Selected landing pad cell lines with and without MAR were maintained in 6‐well plates over 90 generations without antibiotic selection at 37 °C, 10% CO_2_ at 120 rpm. Cells were passaged every 3–4 days and analyzed every 2 weeks by flow cytometry to test fluorescence intensity and percentage of EGFP+/DsRed+ cells.

### 2.4 Generation of stable msAb transfectants

The monospecific antibody expressing stable cell lines were generated by transfecting stable LP clones with Donor_Light, Donor_Heavy and pCAG‐NLS‐HA‐BxB1 (Addgene plasmid # 51271; RRID:Addgene_51,271) vectors. 2 × 10^5^ cells were transfected with 500 ng BxB1 expression plasmid and 1.5 μg of donor vectors (ratio 1:1 between Donor_Light and Donor_Heavy), using the same parameters described above. After transfection, cells were transferred in 12‐well plates in 1 mL of CD CHO supplemented with 8 mM of L‐glutamine and incubated at 37 °C, 10% CO_2_, 120 rpm. Three days after transfection, cells were subjected to double antibiotic selection with 20 μg/mL of puromycin and 600 μg/mL of hygromycin. Integration of both GOIs into the landing pad was monitored by following the loss of fluorescence. After 2 weeks of selection, clonal selection was carried out by plating cells in semi‐solid medium. After 10 days, single colonies were picked from semi‐solid medium and transferred in 96‐well plates. Clones were screened for antibody production by dot‐blot. MsAb producing clones were transferred and maintained in 6 well plates over 90 generations without antibiotic selection. Cells were passaged every 3–4 days and analyzed every 20 generations for msAb production in 30 mL fed‐batch cultures.

### 2.5 Generation of stable bsAb transfectants

BsAb‐expressing stable cell lines were generated by transfecting LP stable clones with Donor_cLight, Donor_KHeavy, Donor_HHeavy, and pCAG‐NLS‐HA‐BxB1 vectors. For transfection, an equimolar mix of donor vectors was mixed with the BxB1 expression vector in a 1:3 ratio (recombinase: payloads) and 2 × 10^5^ cells were transfected with 4 μg of total DNA mixture, using the same parameters described above. After transfection, cells were transferred in 12‐well plates in 1 mL of CD CHO supplemented with 8 mM L‐glutamine and incubated at 37 °C, 10% CO_2_, 120 rpm. After 3 days from transfection, cells were subjected to double antibiotic selection with 10 μg/mL of puromycin and 600 μg/mL of hygromycin. Clones were isolated from semi‐solid medium and screened by dot‐blot using a HRP‐conjugated mouse anti‐Strep‐Tag Classic detection antibody (Bio‐Rad) for the detection of knob heavy chain, HRP‐conjugated His‐Tag monoclonal antibody (Proteintech) for the detection of hole heavy chain and Peroxidase‐AffiniPure Donkey Anti‐Human IgG (H+L) (JK ImmunoResearch) for the detection of the whole antibody.

### 2.6 Clonal selection in semi‐solid medium

To select single clone derived colonies, stable cell pools obtained after antibiotic selection were transferred into semi‐solid medium. Approximately 300 cells were transferred into CHO Growth A medium (Molecular Devices) supplemented with 8 mM L‐glutamine. Cells were incubated at 37 °C, 5% CO_2_, 100% humidity for 10–12 days. Single cell colonies were transferred in liquid CD CHO supplemented with 8 mM L‐glutamine in 96‐well plate and, after recovery, expanded and maintained at 37 °C, 10% CO_2_.

### 2.7 Flow cytometry and single cell‐cloning

Cell fluorescence measurements were performed using a MACSQuant® Analyzer 16 flow cytometer (Miltenyi Biotec, 488 nm laser and 525/50 nm (B1) filter for EGFP detection 488 nm laser and 579/34 nm (B2) filter for DsRed, respectively. Empty CHO‐S cells were used for setting morphological gates to distinguish between double negative, single‐positive (EGFP+ or DsRed+) or double‐positive cells (EGFP+ and DsRed+). Double positive cells were isolated and sorted utilizing a BD FACS Aria III (BD Biosciences, San Jose, CA) using 488 nm laser and 530/30 nm filter for EGFP detection, and 562 nm laser and 585/12 nm filter for DsRed detection. Data analyses were conducted in BD FACSDiva V8.0 software and FlowJo v10.6.2 software. Cells were sorted in 96‐well plates (in 180 μL of CD CHO with 8 mM L‐glutamine). After recovery, they were expanded and maintained at 37 °C, 10% CO_2_.

### 2.8 Stability studies

Selected landing pad cell lines with and without MAR were maintained in 6 well plates over 90 generations in CD CHO without antibiotic selection at 37 °C, 10% CO_2_ at 120 rpm. Cells were passaged every 3–4 days and analyzed every 2 weeks by flow cytometry to test fluorescence intensity and percentage of EGFP+/DsRed+ cells.

Selected msAb producing cells lines were continuously propagated over 90 generations in 6‐well plates without antibiotic selection and subsequently used to inoculate 125 mL shake flask for fed‐batch cultures in 30 mL CD CHO supplemented with 8 mM L‐glutamine at 2 × 10^5^ cells/mL. Fed‐batches were repeated every 20 generations to test antibody titer, viable cell density (VCD) and cell viability.

### 2.9 Genomic PCR amplification of targeted regions

Targeted integration was verified by PCR on genomic DNA using primers binding outside the recombination site into the Landing Pad and primers specific for the gene of interest. For the extraction of genomic DNA, cells were collected by centrifugation and the extraction was performed with DNeasy Blood & Tissue Kit (Qiagen). PCR was carried out using Q5 High‐Fidelity 2× Master Mix and 250 ng of genomic DNA as template in a 25 μL reaction. Thermocycling conditions: 98 °C for 30 s; 30×: 98 °C for 10 s, 72° for 1 min and 20 s; 72 °C for 2 min. Primers are listed in Table S7 (Supporting Information).

### 2.10 Transcript level analysis

RNA was isolated from stable msAb producing clones using the RNeasy mini kit (Qiagen) following the manufacturer's instruction. RT‐qPCR was performed on the Rotor‐Gene Q machine (Qiagen) using Rotor‐Gene Multiplex Rt‐PCR kit (Qiagen) in a triplex assay (two GOIs and one control). The thermal conditions were 15 min at 50 °C and 5 min at 95 °C for reverse transcription step and PCR initial activation step followed by 45 cycles of 15 s at 95 °C and 15 s at 60 °C. Relative expression of HC and LC were calculated using delta Ct analysis method. The expression of eEF1α1 housekeeping gene was used as reference to normalize different RNA samples in each reaction. Each experiment included a “no template control” and each sample was tested in triplicate. Primers and probes are listed in Table S7 (Supporting Information).

### 2.11 Determination of Gene Copy Number

DNA was isolated from stable clones using the DNeasy Blood & Tissue Kit (Qiagen) following the manufacturer's instruction. Fifty nanograms of genomic DNA were used to calculate the transgene copy number using QuantiTect Multiplex PCR Kit (Qiagen) and Rotor‐Gene Q machine. Thermocycling conditions were set up as suggested by the supplier and GCNs were calculated as described by Karlen et al. using Rotor‐Gene Q Series Software.^44^ Sequences of probes for GOIs and reference gene (Beta‐2 microglobulin) as well as primers are listed in Table S7 (Supporting Information). Each experiment included a “no template control” and each sample was tested in triplicate.

### 2.12 Fluorescence in situ hybridization analyses

Exponentially‐growing cells were used to prepare metaphase chromosome spreading for fluorescent in situ hybridization as described by Girod et al.^21^, ^45^ Cells were treated with colcemide for 1 h at a concentration of 0.1 μg/mL and then subjected to hypotonic shock (0.075 M of KCl solution) for 20 min at 37 °C. Subsequently, cells were fixed in 3:1 methanol/acetic acid solution. FISH was performed using fluorescent probes generated by PCR using HighFidelity Fluorescein/Orange PCR Labeling kit (Jena Bioscience). Probes were generated by PCR using pLP_EGFP, pLP_DsRed, pDonor_H and pDonor_L as template utilizing supplier instruction. Utilized primers are summarized in Table S7 (Supporting Information). Chromosomes were counterstained with DAPI. Images were recorded on Cytation5 Imager (Biotek) using 20× objective.

### 2.13 Protein sample preparation and immunoblotting

SDS‐PAGE was performed utilizing Mini‐PROTEAN TGX precast gels (Bio‐Rad). Samples were prepared by mixing them with Laemmli Sample Buffer 4× (Bio‐Rad) either with 10% of 2‐mercaptoethanol (reducing) or without (non‐reducing). Samples were incubated at 95 °C for 5 min and then loaded on the gel. Proteins were transferred to Trans‐Blot Turbo Mini PVDF membranes (Bio‐Rad) using the Trans‐Blot Turbo Transfer System (BioRad). For dot‐blot analysis, Amersham™ Protran® Western blotting membranes (Cytiva) were used to assemble a dot blot manifold and 100 μL of culture supernatant were added in each well. The membrane was blocked using 5% NonFat Milk powder (PanReac, AppliChem) in TBS, then incubated with the primary detection antibody. After three washes in TTBS, bound detection antibody was visualized using Clarity Western ECL Substrate (Bio‐Rad) using Chemidoc gel imaging system (Bio‐Rad). For msAb samples, Peroxidase‐AffiniPure Donkey Anti‐Human IgG (H+L) (JK ImmunoResearch) was used as detection antibody. For BsAb samples, three different immunoblots were prepared that were incubated with different detection antibodies: mouse anti‐Strep‐Tag Classic:HRP detection antibody (Bio‐Rad) for the detection of the knob heavy chain, HRP‐conjugated His‐Tag monoclonal antibody (Proteintech) for the detection of the hole heavy chain and Peroxidase‐AffiniPure Donkey Anti‐Human IgG (H+L) (JK ImmunoResearch) for the detection of the full‐length antibody. Bispecific antibody was purified for SDS‐PAGE and western blot by using Strep‐Tactin®XT Spin Column (Iba lifescience) and His60 Ni Gravity Columns (Takara Bio Company). Buffer exchange of purified bsAb samples to PBS was performed with Amicon Ultra 0.5 mL Centrifugal Filters Ultracel ‐10 K (Millipore).

## 3 RESULTS

### 3.1 Generation of master host cell line containing Landing Pad

**FIGURE 1:**
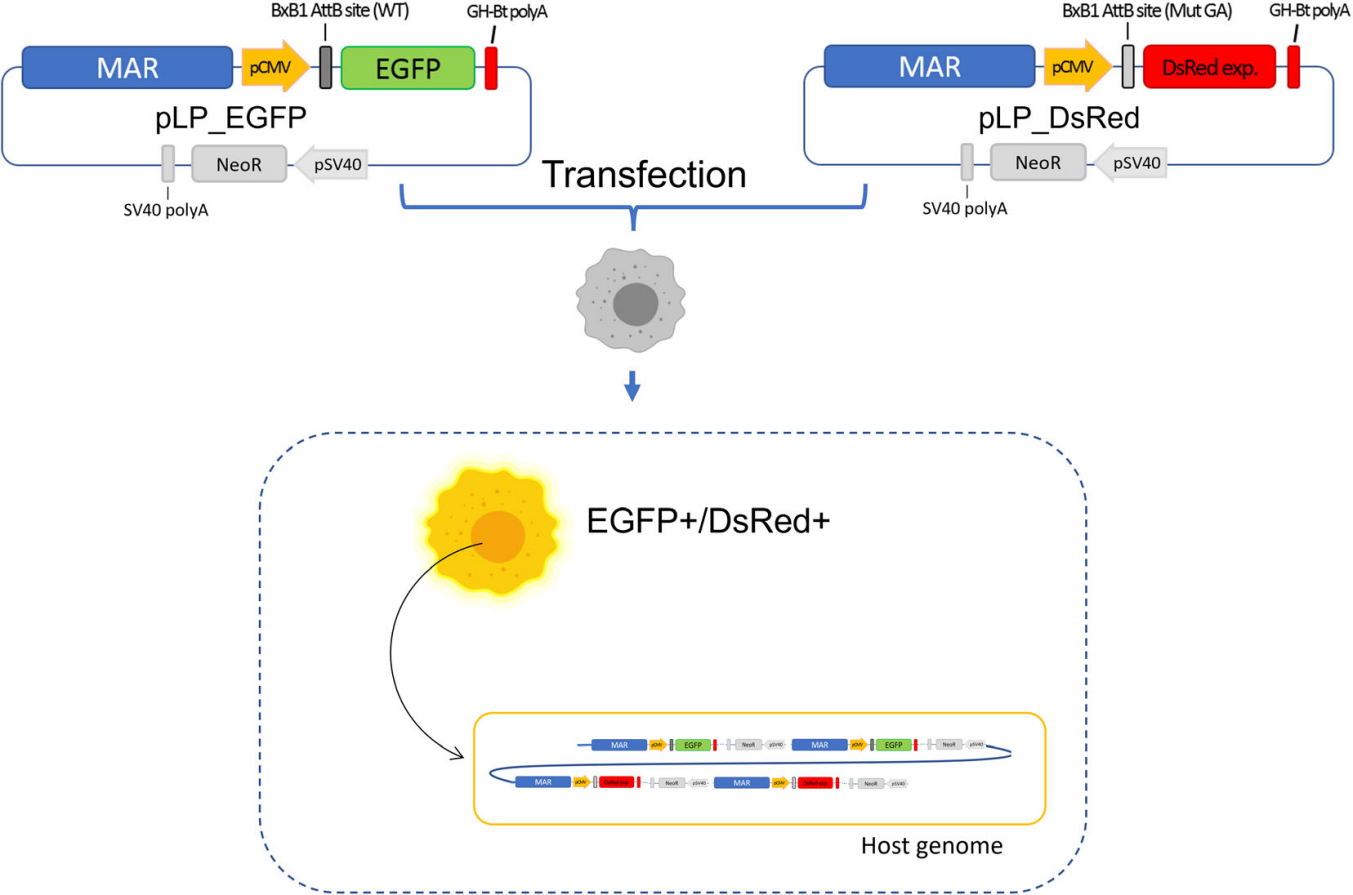
Schematic representation of the generation of LPs‐cMAR containing cells. Cells were transfected with the landing pad vectors harboring the 5′ chicken lysozyme MAR and then selected by G418 selection and/or by sorting double positive fluorescent cells. pLP_EGFP vector contains the chicken 5′ lysozyme MAR, pCMV, AttB‐WT site for BxB1 recombinase, EGFP reporter gene, and a ghb polyA tail. pLP_DsRed vector contains the chicken 5′ lysozyme MAR, pCMV, AttB‐GA (mutated) site for BxB1 recombinase, DsRed reporter gene, and a ghb polyA tail. In addition, both vectors contain the Neomicyn resistance cassette (pSV40‐NeoR‐SV40polyA) for G418 antibiotic selection

Targeted integration of genes of interest (GOIs) by recombinase‐based landing pad system require two fundamental steps: generation and selection of host cell clones harboring the landing pad and generation of stable integrants by recombinase‐mediated integration of GOIs into the LP. To generate a host cell line containing MAR‐rich landing pads, CHO‐S cells have been transfected with two different landing pad vectors. The landing pad itself consists of the chicken lysozyme 5′ MAR (cMAR) followed by a recombination site for BxB1 located between a strong promoter (pCMV) and a reporter gene to support the promoter trap strategy (Figure 1). Two different LP vectors have been created during this study to establish a double landing pad system, which can be individually addressed for orthogonal integration of two GOIs. One landing pad vector contains the cMAR sequence followed by a pCMV promoter, the AttB wild type site for BxB1 recombinase, EGFP reporter gene and a bhg polyA tail. The other landing pad vector harbors the same cMAR sequence followed by pCMV promoter, the AttB site for BxB1 recombinase containing a central dinucleotide mutation (GA instead of GT), DsRed reporter gene and a bhg polyA tail. The use of the MAR in this construct should help the integration of multiple copies of the landing pad into the host genome via concatemer formation, protect it from silencing due to position effects and improve the stability of the system.^19^, ^46^ The orthogonal AttB sites for BxB1 can recombine only with the AttP sites carrying the matching central dinucleotide, allowing integration of GOI specifically at one or the other landing pad. The dual reporter gene system allowed the direct visualization of the landing pad integration into the host genome by testing cell fluorescence for DsRed and EGFP, which can be used as a parallel selection strategy additionally to G418 selection.

**FIGURE 2:**
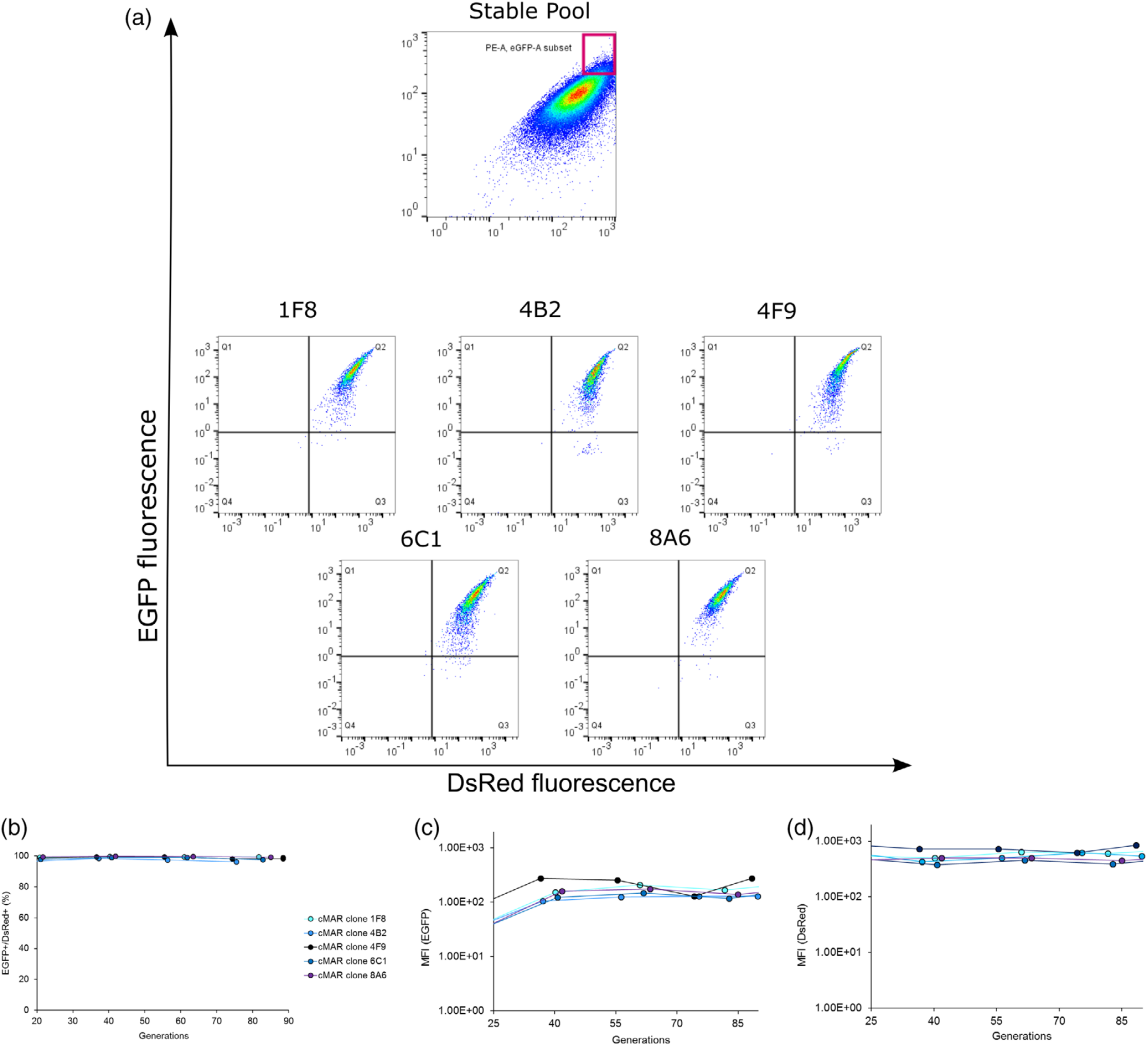
Flow cytometer analysis (FCA) of selected LPs_cMAR clones and stability test. (a) FCA plots for the analysis of EGFP and DsRed levels on pool or single clone level. Gating strategy for cell sorting and cell isolation depicted in red. Gating strategy was defined using untransfected CHO‐S as negative control. The fluorescence for single clones was tested at generation 20. The EGFP+/DsRed+ subpopulation is shown in gate Q2. B‐D) Stability test representing the frequency of EGFP+/DsRed+ clones (b) and median fluorescence intensity (MFI) of the LPs_cMAR clones for EGFP (c) and DsRed (d)

**FIGURE 3:**
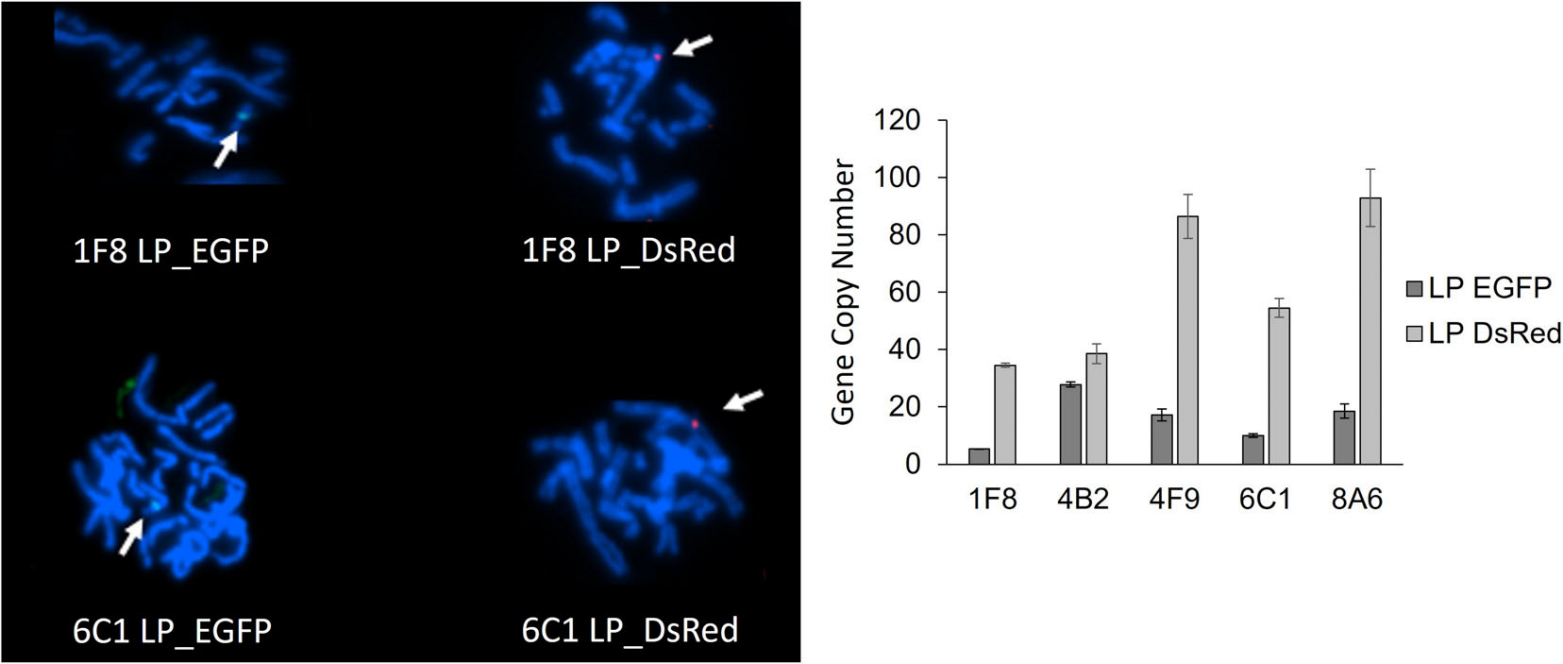
Characterization of landing pad integration and landing pad copy number in LPs_cMAR clones. (a) FISH analyses on metaphase chromosomes of clones 1F8 and 6C1. Chromosome were hybridized with FITC‐labeled probe against LP_EGFP and ATTO594‐/AF594‐/Texas Red‐labeled probe against LP_DsRed. (b) Gene copy number analysis of LP_EGFP and LP_DsRed showing mean and standard deviation (SD) for technical replicates. GCN were calculated as described by Karlen et al. using Rotor‐Gene Q Series Software

Cells were consecutively transfected with two times 2 μg of pLP_DsRed with a recovery period of 21 h between both transfections. After 2 weeks of G418 selection and semi‐solid plating, a stable pool with DsRed positive cells was isolated. Cells were then transfected with pLP_EGFP, subjected to geneticin selection and plated in semi‐solid medium to select an enriched pool of cells positive for EGFP as well as DsRed. Double‐positive clones (EGFP+/ DsRed+) were isolated by FACS (Figure 2a). For further investigations, we selected the five clones with the highest fluorescence levels for both EGFP and DsRed. These clones have been expanded and maintained in culture to test fluorescence properties, growth in batch culture, gene copy numbers (GCN), fluorescence in‐situ hybridization (FISH) and their genetic stability. Clones were propagated over 90 generations without antibiotic selection, and they were tested every ∼20 generations for EGFP and DsRed expression. All five clones containing MAR‐rich landing pads (LPs‐cMAR) showed a homogeneous double‐positive population (≥96% of the total cell population), which remained stable over the tested time period without any drop neither in median fluorescence intensity nor in percentage of double‐positive cells (Figure 2b). To evaluate if LPs integrated at single or multiple chromosomal sites into the host genome, chromosome metaphases of clones 1F8 and 6C1 were prepared and hybridized with fluorescent probes. All tested samples showed a single integration site for both EGFP and DsRed containing landing pads (Figure 3a). Analysis of gene copy number (GCN) revealed differences between clones in terms of LP copy number and LPs ratio even if little variation was registered for EGFP and DsRed fluorescence intensity. All clones showed 1.4‐7‐fold higher copy numbers for LP_DsRed in comparison to LP_EGFP. The highest ratio of LP_DsRed in comparison to LP_EGFP was recorded for 1F8, whereas 4B2 only showed a 1.4‐fold increased GCN for LP_DsRed. Clones 4F9, 6C1 and 8A6 showed a similar LP_DsRed/LP_EGFP ratio of 5:1 (Table S1, Supporting Information). This difference in copy number is probably due to the two consecutive transfection with pLP_DsRed.

**FIGURE 4:**
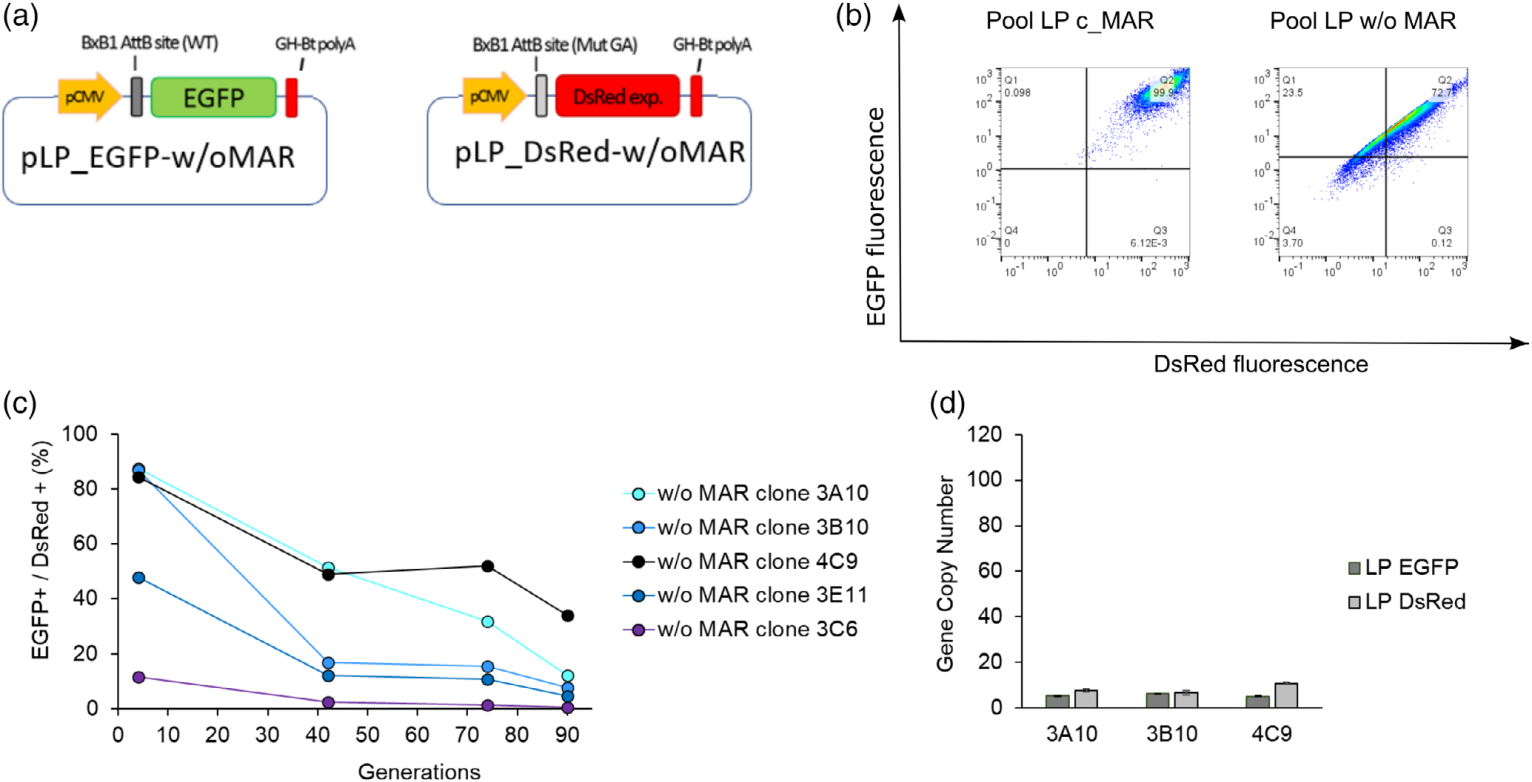
Generation and characterization of LP_w/o‐MAR clones. (a) LP vectors without MAR. These vectors contain the same features as LP_cMAR vectors except for the 5′ chicken lysozyme MAR. (b) Flow cytometer analyses of cell pools containing MAR or no MAR sequences, respectively. Q2 quadrant represents the double positive (EGFP+/DsRed+) population. (c) Stability analysis of LP_w/o‐MAR clones. Clones were tested by flow cytometry to assess the percentage of double positive cells over 90 generations. (d) Gene copy number analysis of LP_EGFP‐w/oMAR and LP_DsRed‐w/oMAR showing mean and standard deviation (SD) for three technical replicates. Samples were analyzed at generation 40

To assess the effect of the chicken lysozyme 5′ MAR sequence in the landing pad, control cell lines without MAR have been generated. CHO‐S cells were transfected with pLP_w/o‐MAR vectors (Figure 4a) and a stable pool and clones were selected for further analyses. The polyclonal population of LP_w/o‐MAR cell showed 72.7% of double‐positive cells, indicating a less successful integration of LP‐vectors in comparison to the MAR‐containing constructs, which resulted in 92.7% double‐positive cells. After clonal selection, five clones were expanded and maintained in culture to characterize them and evaluate their genetic stability. Three out of five clones (3A10, 3B10 and 4C9) showed high homogenous EGFP and DsRed fluorescence and more than 80% double‐positive (DP) cells (Figure S1, Supporting Information). Stability tests revealed a significant drop in DP population for all tested clones. The fluorescence level of clone 3B10 registered the strongest drop as early as after 40 generations. At generation 90, all clones showed a 40% reduced fluorescence signal for both reporter proteins. To investigate whether differences in EGFP/DsRed expression were linked to the number of copies of integrated landing pads, GCN analyses were repeated for clones 3A10, 3B10 and 4C9. The copy number for both landing pads was lower than previously reported for the MAR‐containing cells (ranging between 5–6 copies and 6–10 copies for LP_EGFP and LP_DsRed, respectively) and the ratio between LP_DsRed and LP_EGFP varied between 1:1 and 1:2 (Table S1, Supporting Information). Despite differences in landing pad copy numbers and inclusion of MAR element into the LPs, all the clones analyzed showed similar growth curves when cultured in batch mode (Figure S1, Supporting Information). Generated clones containing LPs_cMAR were subsequently used to perform site‐specific integration of donor vectors for the generation of msAb‐producing cell lines. Due to the early loss of fluorescence for LP_w/o‐MAR cells, those clones were not selected for site‐specific integration (SSI) step.

### 3.2 Generation of msAb expressing cell line by SSI using BxB1 system

**FIGURE 5:**
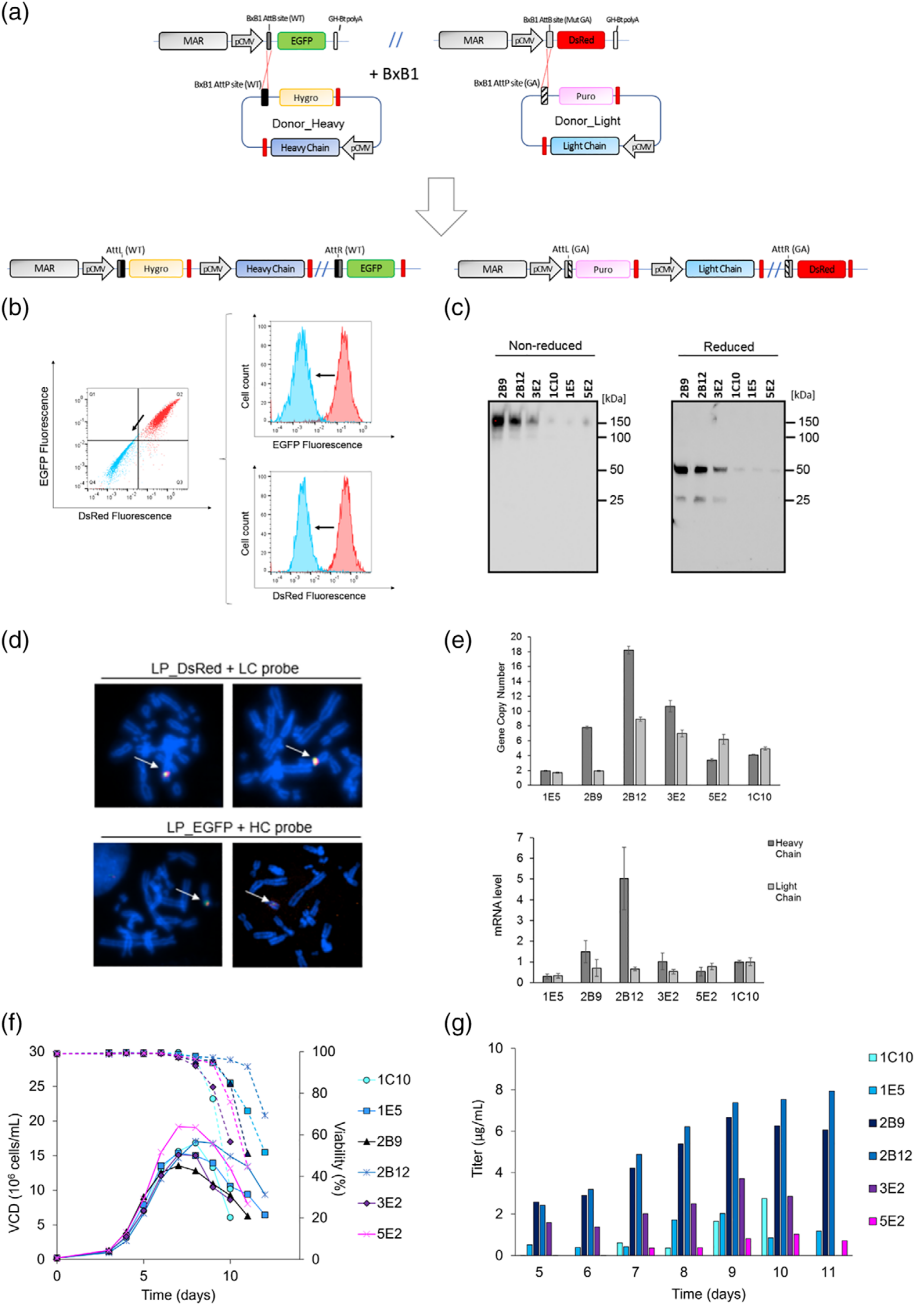
Generation and characterization of msAb producing cell lines. (a) Schematic representation of the integration of the donor vectors containing HC and LC genes into LP_EGFP and LP_DsRed, respectively. (b) Analysis of cell fluorescence by flow cytometry for LP clone 8A6 before (red) and after SSI (blue) of donor vectors. Flow cytometry diagrams represent a shift from EGFP+/DsRed+ to EGFP‐/DsRed‐ pool which correspond to integration of both donor vectors into the respective LP. (c) Western Blot with anti‐Human IgG (H+L) analyses of fed batch supernatants for six cell lines under non‐reduced (left) and reduced (right) conditions. Ten microliters of supernatant, collected on harvest day, were loaded on each lane (medium: CDCHO; feeding strategy: 1% Hyclone Feed A and 0.5% Hyclone Feed B, daily feeding from day 3). (d) FISH analyses using probes against LP_DsRed and the light chain gene (upper panel) or LP_EGFP and the heavy chain gene (lower panel), respectively. Probes against LP_DsRed and the heavy chain gene were labeled with ATTO594‐/AF594‐/Texas Red‐dUTP; Probes against LP_EGFP and the light chain gene were labeled with FITC‐dUTP. (e) GCN analysis (upper chart) and mRNA level (lower chart) for msAb expressing clones, showing the mean and the SD for technical replicates. Dark gray bars represent heavy chain copy number/mRNA level, light gray bars represent light chain copy number/mRNA level. Values are relative to the expression levels of clone 1C10 and normalized to the housekeeping gene eEF1A1. (f,g) Results of fed‐batch cultures of msAb expressing clones in CD‐CHO. Cells were fed with 1% Hyclone Feed A and 0.5% Hyclone Feed B, daily feeding from day 3. Solid lines represent the viable cell density (VCD); dotted lines represent the cell viability. Bar charts (on the left) represent antibody titer measurements performed with protein A biosensors via BLI

To test the generated host cell lines for BxB1 site‐specific integration, we constructed two donor vectors for the expression of msAb‐Fer a human monoclonal antibody developed by Ferring (Figure 5a). The heavy chain (HC) gene was inserted into a modified pD607 vector containing the BxB1 AttP‐WT recombination site followed by the promotor‐less hygromycin selection marker (donor_heavy), as described in the material and method section. The light chain (LC) gene was inserted into a modified pD609 vector containing the mutated BxB1 AttP‐GA recombination site followed by the promotor‐less puromycin selection marker (donor_light). The generated donor vectors contain orthogonal AttP recombination sites which can recombine only with the matching AttB site contained in the landing pads. After recombination, the donor vectors will be fully integrated into the corresponding landing pad causing the shift of the reporter gene from its promoter (provoking the loss of fluorescence) and the integration of the selection marker close to the pCMV. Donor_heavy vector containing AttP‐WT sites will be integrated only into LP_EGFP (containing AttB‐WT site), causing loss of EGFP fluorescence and expression of the hygromycin resistance gene. Donor_light vector containing AttP‐GA sites will be integrated only into LP_DsRed (containing AttB‐GA site), causing loss of DsRed fluorescence and expression of the puromycin resistance gene. Generated donor vectors were co‐transfected with BxB1 expressing vector into host cells to test the simultaneous site‐specific integration into both landing pads. For this purpose, LPs_cMAR clones 1F8, 4B2, 6C1 and 8A6 were co‐transfected with the two donor vectors and with pCAG–NLS–HA‐BxB1 (Addgene #51271) vector containing the BxB1 recombinase gene. Cells were maintained under double antibiotic selection for 10–12 days and then cells were plated in semi‐solid medium for clone isolation. Selected clones showed the loss of both fluorescence markers confirming the success of GOI integration into the LPs (Figure 5b). After screening for antibody expression by dot blot (Figure S3, Supporting Information), six productive clones were selected and tested for msAb production in 30 mL fed‐batch cultures. Supernatants from day 7 of cultivation were resolved under denaturing and non‐denaturing conditions by SDS PAGE and then transferred to a nitrocellulose membrane for western blot (WB) analysis. Clones 2B9, 2B12 and 3E2 showed the expected bands at 50 kDa and 25 kDa for HC and LC, respectively, under denaturing conditions. A strong signal at 150 kDa was detected for these samples under non‐reducing conditions, indicating the formation of intact antibody molecules. In addition, a faint band at 100 kDa is visible, probably representing heavy chain dimers. Supernatants from samples 1C10, 1E5 and 5E2 showed only a faint band at 50 kDa under denaturing conditions. In addition, a faint band at 150 kDa for these samples under non‐reducing conditions suggests the presence of full‐length antibody molecules.

### 3.3 Characterization of msAb expressing clones

To further characterize the selected msAb expressing clones, FISH analysis, GCN analysis, mRNA level analysis and genetic stability tests were performed. To show that the integration of HC and LC genes happen specifically into LP_EGFP and LP_DsRed, respectively, colocalization of orthogonal probes was performed in a FISH assay on metaphase chromosomes of clone 2B9. A single integration site has been highlighted for both HC and LC gene copies, which corresponds to the matching landing pad site (Figure 5d), showing no random integration events for these genes in the selected analyzed clone. Due to the high number of LP copies integrated into the genome of each host cell, HC and LC copy numbers for each productive clone were tested and compared with the LP copy numbers of the corresponding progenitor. The analyzed clones were heterogeneous in terms of GOI copy number and HC:LC ratio (Table S2, Supporting Information). In addition, clones 1C10 and 1E5, which derived from the same host cell progenitor (LP clone 1F8) showed a HC:LC ratio of 0.8 and 1.1, respectively. Clones 2B9, 2B12 and 3E2, which derived from LP clone 8A6, showed a HC:LC ratio of 4.1, 2, and 1.5, respectively (Table S2, Supporting Information). These differences between clones could be partially explained by the high number of LP sites available for GOI integration and non‐optimized transfection parameters that could lead to an incomplete occupancy of LP sites by the donor vectors. This hypothesis was confirmed by PCR on genomic DNA using primers flanking the integration site for the GOIs (Figure S2, Supporting Information) which showed the presence of unoccupied LP_DsRed and LP_EGFP sites. However, LP_EGFP sites showed a higher occupancy than LP_DsRed sites (Table S2, Supporting Information). To assess whether the differences in GOI copy number were reflected on mRNA level and antibody production, fed‐batch cultures and qRT‐PCR analyses were done to analyze HC and LC mRNA expression levels. Values are relative to the expression levels of clone 1C10 and normalized to the housekeeping gene eEF1A1. All clones displayed a direct correlation between HC copy number and its mRNA level (Figure 5e). However, the same correlation was not found for LC copy number and its transcript level, supporting the hypothesis that msAb‐Fer9 was a hard‐to‐express antibody. Fed‐batch cultures showed similar growth for all the six clones tested, with clone 2B9, 2B12, and 3E2 reaching the highest final titer (Figure 5f,g). For these clones, which derived from the same MHC progenitor, the final antibody titer correlated to the HC mRNA level. Clones 1E5 and 5E2 showed lower HC/LC mRNA level resulting in lower antibody titers. Interestingly, mRNA levels of HC and LC for clone 3E2 were lower compared to clone 1C10. However, quantification revealed a fourfold increased antibody titer for 3E2 suggesting potential clone‐to‐clone variation and difficulties in the expression of msAb antibody sequence. To test antibody production and stability, clones were routinely propagated over 85 generations and used to seed 20 mL cultures at 2 × 10^5^ cells/mL, cultured in fed‐batch mode, every 20–30 generations. None of the clones showed a drop in antibody titer or decreased growth rate (Figure S2 F‐G, Supporting Information), proving that BxB1 site‐specific integration (SSI) system resulted in stable integration of GOIs over time and in stable expression from both LP sites of HC and LC.

### 3.4 Generation of bsAb expressing cell line by SSI using BxB1 system

**FIGURE 6:**
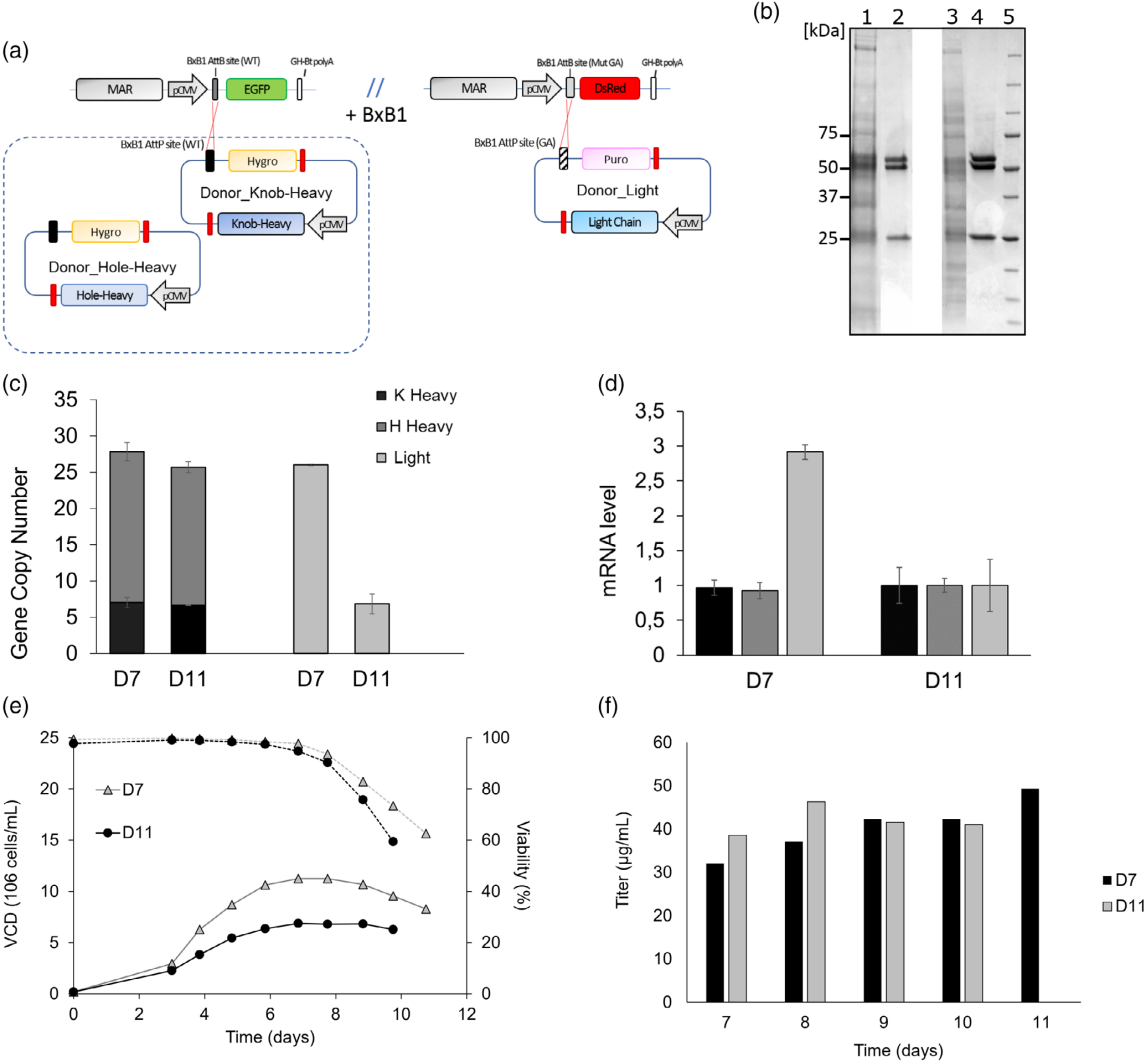
Generation of bsAb production cell lines. (a) Schematic overview of the integration of donor vectors containing kHC/hHC and cLC genes into LP_EGFP and LP_DsRed, respectively. (b) SDS‐PAGE analysis of unpurified supernatant (harvest day) and purified bsAb from clone D7 (lane 1 and 2), unpurified supernatant (harvest day) and purified bsAb from clone D11 (lane 3 and 4). (c,d) Copy number analyses and mRNA level for bsAb expressing clones showing the mean and the SD for three technical replicates. (e,f) Results of fed‐batch cultures of bsAb expressing clones in CD‐CHO. Cells were fed with 1% Hyclone Feed A and 0.5% Hyclone Feed B, daily feeding starting on day 3. Solid lines represent viable cell density (VCD); dotted lines represent viability; D7 and D11 clones are indicated as triangle or solid circle points respectively. Bar charts (on the left) represent antibody titer measured with Octet K2 system with protein A biosensors

To test the feasibility of the developed TI approach for complex recombinant protein expression, LP‐bearing host cell lines were tested for the production of a humanized chicken bispecific antibody (bsAb‐Fer). BsAb‐Fer is composed of two identical common LCs and two different HCs, which have been engineered for knob‐into‐hole pairing to promote heterodimerization.^47^, ^48^ In addition, knob and hole HCs (referred to as kHC and hHC) have been modified to contain a Twin‐Strep‐tag and His‐tag, respectively, to facilitate purification and homo−/hetero‐dimer identification. Three different donor vectors were generated using the three genes coding for the bsAb. HC cassettes were subcloned into the donor vector containing a hygromycin gene and AttP‐WT recombination site, thus both donor‐kHC and donor‐hHC targeted LP_EGFP and their integration could be monitored by loss of EGFP fluorescence (Figure 6a). The common LC was subcloned into a donor vector containing puromycin and AttP‐GA site and its integration into LP_DsRed could be monitored by loss of red fluorescence. Different ratios of donor vectors (kHC:hHC:LC) were tested for transfection into LP‐bearing host cell lines 4B2, 6C1 and 8A6: 1:1:1, 1:1:2, 2:1:1, 1:2:1. After selecting a stable pool using double antibiotic selection, single cell‐derived colonies were picked from semi‐solid medium and screened for heterodimer production by dot‐blot assay using anti‐hIgG (H+L), anti‐His‐tag and anti‐Strep‐tag detection antibody (Figure S4, Supporting Information). After clonal screening and analyses by SDS‐PAGE and WB, two clones (D7 and D11) showed bands for kHC, hHC, and LC and were subsequently selected for further experiments. Both clones derived from the same LP progenitor (clone 4B2) transfected with 1:1:1 molar ratio of donor vectors (kHC:hHC:LC). Analysis of gene copy number showed that the two selected clones contained a similar copy number and proportion for kHC and hHC, which occupied >98% of LP_EGFP sites available in the progenitor genome (Table S3, Supporting Information). However, the common LC gene copy number varied more, with clone D7 containing 3.8 times more LC gene copies than clone D11 and an occupancy rate of LP_DsRed of 67.4% (Figure 6b, Table S3, Supporting Information). Differences in gene copy number were reflected on mRNA level, with clone D7 showing 2.9‐fold increased mRNA levels in comparison with D11 (Figure 6c,d). In addition, these clones were tested in fed‐batch cultures and further analyzed for bsAb production. Both clones showed similar behavior in culture, with clone D7 reaching the higher VCD (1.6 times higher than clone D7) and longer culture duration. Despite differences in LC expression and VCD, clones displayed similar final titers of ∼40 μg/mL at day 10 (Figure 6e). These results indicate the feasibility to use LP‐bearing cell line generated in this study for simultaneous expression of more than two different genes and the efficient development of stable recombinant cell lines able to produce intact complex molecules.

### 3.5 Fed‐batch cultures and media test for msAb and bsAb cell lines

**FIGURE 7:**
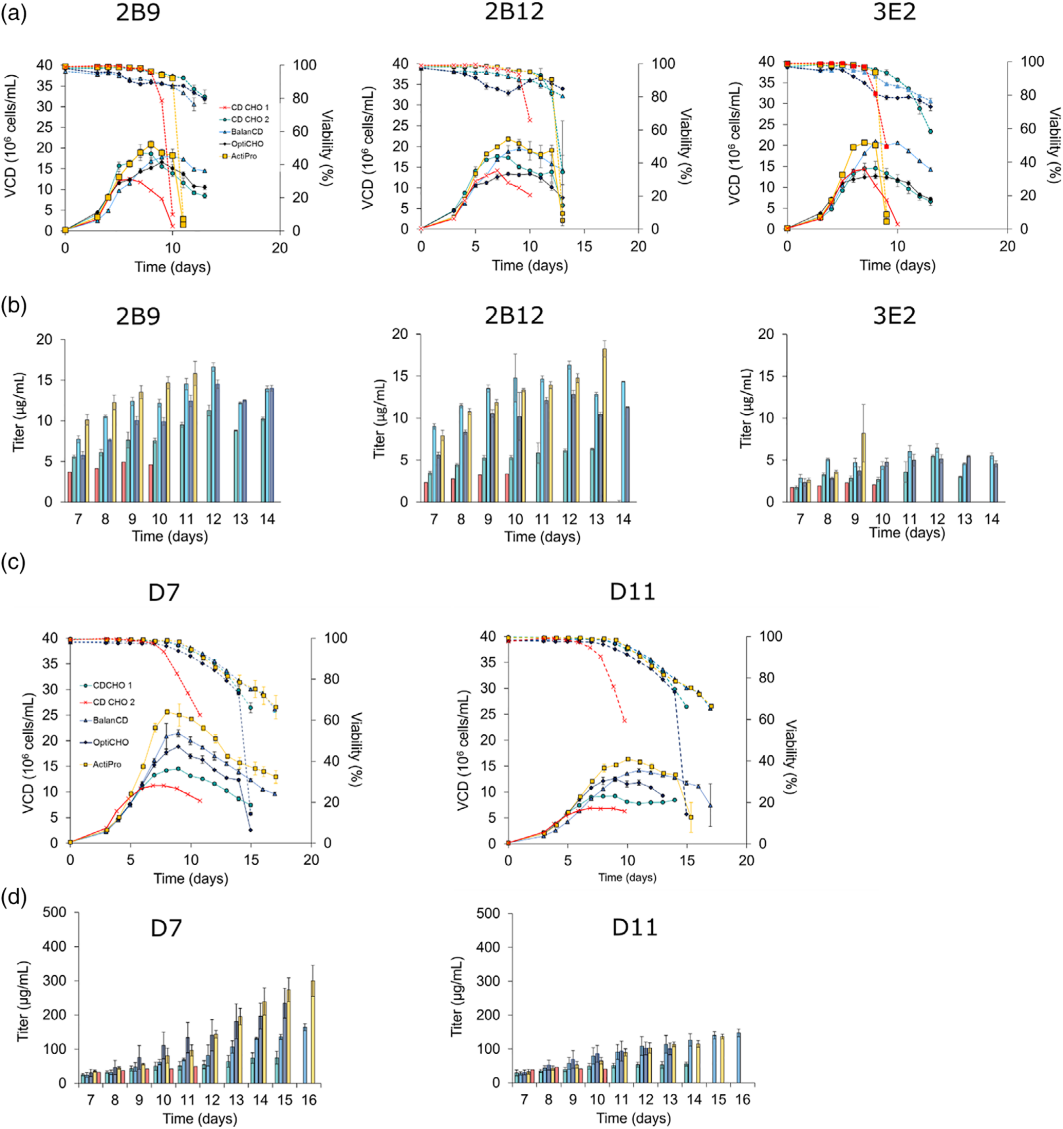
Fed‐batch cultures and media test for msAb and bsAb cell lines. (a,b) Fed‐batch cultures for msAb expressing clones 2B9, 2B12 and 3E2. Solid lines represent Viable Cell Density (VCD); dotted lines represent viability; bar charts represent antibody titer measured with Octet K2 system with protein A biosensors. Error bars represent SD of biological triplicates. Cells were fed with 2% Hyclone Feed A and 0.2% Hyclone Feed B, daily feeding from day 3. As comparison, CD CHO 1 was daily fed with 1% Hyclone Feed A and 0.5% Hyclone Feed B starting on day 3. (c,d) Fed‐batch cultures for bsAb expressing clones D7 and D11. Solid lines represent Viable Cell Density (VCD); dotted lines represent viability; bar charts represent antibody titer measured with Octet K2 system with protein A biosensors. Error bars represent SD of biological triplicates. Cells were fed with 2% Hyclone Feed A and 0.2% Hyclone Feed B, daily feeding from day 3. As comparison, CD CHO 1 was daily fed with 1% Hyclone Feed A and 0.5% Hyclone Feed B starting on day 3

Several studies reported the impact of culture media in cell growth, specific productivity, process duration and final titer.^49^, ^50^, ^51^ In order to improve the culture conditions for the developed recombinant cell lines, we tested four different basal media (CD CHO, Balan‐CD, OptiCHO and ActiPro) in combination with HyClone Feed 7a and HyClone Feed 7b (2% and 0.2%, added daily from day 3). Clone 2B9, 2B12, and 3E2 expressing the monospecific antibody, as well as clones D7 and D11 expressing the bispecific antibody, were cultivated in fed‐batch mode and tested for growth and antibody production. For monospecific clones, all three conditions supported an improvement in VCD and culture duration (Figure 7a). Culture duration was improved up to 4 days compared to the initial fed‐batch strategy (Figure 7a, Table S4, Supporting Information) and the final titer increased up to 5‐fold. All the clones reached highest VCD and highest titer in ActiPro medium. However this medium did not support the longest culture duration. Similar results in terms of culture duration, cell growth and final titer were observed for bsAb‐expressing clones. Culture duration was improved up to 6 days and bsAb final titer was increased up to 7‐fold compared to the initial fed‐batch strategy (Figure 7c, Table S5, Supporting Information). The highest VCD was achieved in Balan‐CD and ActiPro media, which also support the highest harvest titer (Table S5, Supporting Information). Results showed an increase in final titer for both recombinant cell lines expressing monospecific and bispecific antibodies attributable to an improvement in culture conditions compared to the initial fed‐batch strategy, which increased culture duration and maximum VCD suggesting that further culture optimization might enable maximizing the titer and specific productivity.

## 4 DISCUSSION

Recent developments of TI systems relied mainly on the integration of landing pads into pre‐determined hot spots in the host cell genome using molecular tools such as ZNF nucleases, CRISPR‐CAS9 system or lentiviral transduction.^36^, ^41^ Upon identifying and characterizing highly active transcription sites, these systems are limited to integrating a few LP copies.^52^, ^53^ A recent study reported the integration of multiple landing pads in pre‐screened CHO genomic hot spots that were investigated by lentiviral integration and sequencing.^36^ However, the number of integrated landing pads remained limited to three. To increase the number of GOI copies, the authors utilized multi‐gene vectors expressing up to three msAb copies. This approach resulted in a reduction of integration efficiency and difficulties in clone characterization and validation.^36^, ^41^ To investigate the possibility of integrating multiple copies of LPs into the host genome without the use of hard‐to‐engineering genomic tools, we developed a dual LPs system containing a strong epigenetic element, the chicken lysozyme 5′ MAR sequence. This element, included in both landing pad vectors, should facilitate the generation of a genomic “artificial hot‐spot” harboring several copies of the expression vector, each flanked by the MAR sequence, creating de novo a protected, independent chromatin environment for the landing pads.^20^, ^54^ The chicken lysozyme 5′ MAR has been proven to be a potent epigenetic element by reducing variegation effects on integrated transgene(s), enhancing gene expression and increasing transgene copy number co‐integrated at unique chromosomal loci.^46^, ^54^, ^55^, ^56^

After sequential transfections and selection, we selected five stable cMAR_LPs cell lines to test their applicability for recombinant protein expression. The utilized transfection strategy allowed us to successfully obtain clones comprising both LPs. The differences in copy number between landing pads for each clone are attributable to the consecutive transfections with LP_DsRed, which increased cellular DNA uptake and enhanced the probability of transgene integration as shown in previous studies in CHO cells using a MAR element.^46^ FISH analyses showed single integration spots for each landing pad (Figure 3a), supporting the hypothesis that the inclusion of cMAR helped the formation of concatemers and integration of LP copies at one or few chromosomal loci, even in the context of consecutive transfections, according to Grandjean et al.^21^, ^46^ In addition, analyses of gene copy numbers revealed a higher number of LP copies clones containing the chicken 5′ lysozyme MAR compared to clones which did not contain the MAR sequence as well as stronger and stable fluorescence expression for each reporter gene, indicating stable integration of both LPs. All these data suggest that the cMAR not only helps the integration of LP into the genome, increasing the number of copies of LP per cell compared to the control, but it also improves the cells' long‐term genetic stability, as previously proven in other studies.^11^, ^21^, ^46^, ^57^ These positive effects of the MAR element, reasonably due to its role of epigenetic element acting as boundary element and its ability to mediate transgene integration into permissive and active sites of the genome, prevents the elaborate work of identifying genomic hot spots prior to integration.^19^, ^22^, ^54^. However, it will be interesting to have data on LP integration sites by analyzing many more clones to determine if there are some preferential genomic insertion sites.

In the next step, we tested whether the generated LP‐bearing cell lines could be used to integrate light and heavy chain genes for antibody expression. The LP system with its orthogonal BxB1 recombination sites (attP/attB wild type and with GA central mutation) ensured efficient integration of the donor vectors and avoided off‐target integration events due to the very limited crosstalk between the utilized recombination sites.^34^, ^36^, ^58^, ^59^

In addition, the promotor‐trap strategy using donor vectors containing promotor‐less selection markers further reduced the risks of off‐target integration and allowed clone selection by loss of fluorescence and double antibiotic selection, reducing time and resources needed for screening of productive cell lines. Heavy and light chain genes were successfully integrated into LP_EGFP and LP_DsRed, respectively. The developed msAb cell lines reached the maximum harvest titer in Balan‐CD and ActiPro (16–18 μg/mL), which is comparable to titers obtained in previous studies using BxB1 recombinase.^34^, ^36^ The obtained results and low titers for the msAb‐expressing clones might be caused by translational and post‐translational bottlenecks caused by the sequence among other potential causes. To confirm this hypothesis, an msAb with a known expression profil should be tested. In addition, gene copy number analysis as well as the mRNA level analysis for the msAb cell lines showed inconsistent outcomes since the high copy number of the light chain gene did not translate to high mRNA levels.

The bsAb cell lines reached a maximum harvest titer in ActiPro medium (312 μg/mL) comparable to data previously reviewed by Wang et al.^47^ Gene copy number for hHC, kHC and cLC correlate with the mRNA level and antibody titer indicating no translational or post‐translational bottleneck. Despite of that, cell lines generated in this study are not optimized yet and several points can be addressed to improve the system such as transfection conditions in order to find the best DNA amount and ratios for landing pad total occupancy, convert TI to recombinase‐mediated cassette exchange (RMCE) to avoid integration of additional DNA sequences (e.g. ampicillin resistance cassette) and also further optimize culture condition in fed‐batch mode using DOE approach.^26^, ^33^, ^40^, ^49^

In summary, in this work, we developed a multi‐copy MAR‐rich landing pad system for the orthogonal, simultaneous integration of several copies of up to three GOIs. Generated LP‐bearing host cell lines showed high stability and could be considered suitable for GOIs integration and expression. Presence of MAR element helped the integration of several copies of both landing pads and increased system stability compared to a non‐MAR comprising control cell line. The designed system allowed the monitoring of integration of up to two different genes by loss of fluorescence of reporter genes (EGFP and DsRed) and double antibiotic selection. However, integration of more than two genes is also possible as demonstrated by the expression of a bispecific antibody. The developed system helps the selection of stable integrant clones which produce the protein of interest, using low‐cost easy to handle selection methods such as single cell derived colony picking from semi‐solid medium. Having at disposition high throughput system (e.g., Beacon®, ClonePix®) or a FACS for single cell sorting, after TI, timelines for cell line development could be significantly reduced. Despite the benefits, the lack of correletion between GOIs copy number and mRNA level should be further investigated and additional optimization is needed to improve the integration of the GOIs into the landing pads and its expression.

However, the developed system resulted in a significant reduction of time and resources for cell line generation for both monospecific and more complex antibody formats and it represents a further step towards a more efficient and more rapid cell line development process.

## CONFLICT OF INTEREST

The authors declare no conflict of interest.

## AUTHOR CONTRIBUTIONS

**Claudia Oliviero:** Conceptualization (lead); data curation (lead); investigation (lead); methodology (lead); resources (lead); validation (equal); visualization (lead); writing – original draft (lead); writing – review and editing (equal). **Steffen C. Hinz:** Data curation (lead); formal analysis (lead); investigation (equal); methodology (equal); supervision (lead); validation (lead); visualization (lead); writing – review and editing (equal). **Jan Bogen:** Resources (supporting). **Henri Kornmann:** Funding acquisition (equal); project administration (supporting); supervision (supporting); writing – review and editing (supporting). **Björn Hock:** Conceptualization (supporting); funding acquisition (equal); project administration (supporting); supervision (supporting); writing – review and editing (supporting). **Harald Kolmar:** Project administration (supporting); resources (supporting); supervision (supporting); writing – review and editing (supporting). **Gerrit Hagens:** Conceptualization (lead); funding acquisition (lead); project administration (lead); supervision (lead); validation (equal); writing – review and editing (equal).

### PEER REVIEW

The peer review history for this article is available at https://publons.com/publon/10.1002/btpr.3254.

## Supporting information


**Appendix S1**Supporting Information

## Data Availability

Data available on request from the authors
