# Supplementary material for: Generation of a host cell line containing a MAR‐rich landing pad for site‐specific integration and expression of transgenes
Source: Biotechnol Prog. 2022 Apr 25;38(4):e3254. doi: 10.1002/btpr.3254 (PMC9539524; doi:10.1002/btpr.3254)
Supplement: Supplementary file 1 — Appendix S1Supporting Information [file BTPR-38-e3254-s001.docx]

**Supporting Information (SI)**

Generation of Master Host Cell line containing MAR-rich landing pad for transgene site specific integration and expression

**
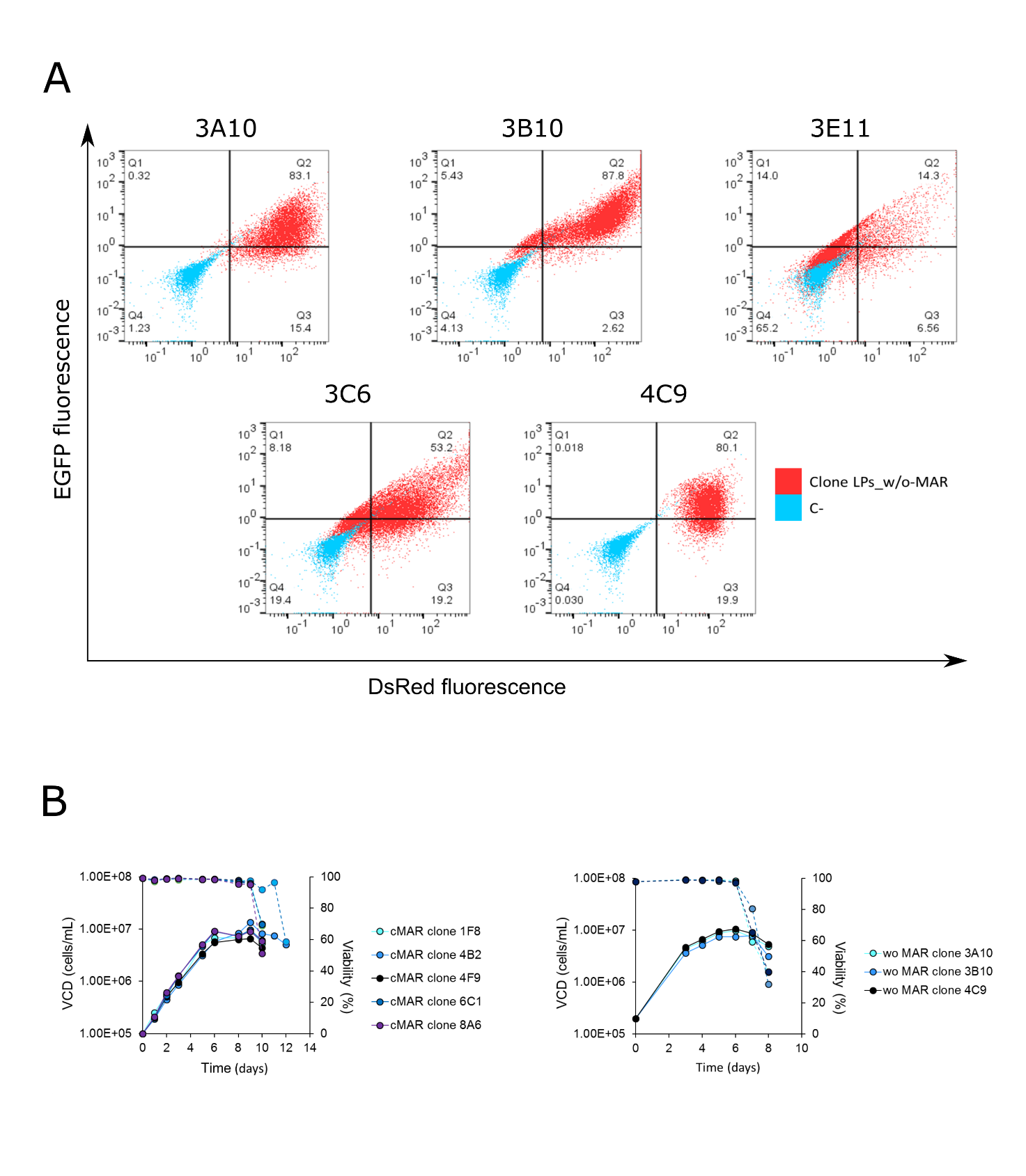
Figure S1.** Fluorescence characterization of LPs_w/o-MAR clones and batch culture for LPs_cMAR and LPs_w/o-MAR clones. **A)** Representation of EGFP and DsRed fluorescence for LPs_w/oMAR clones. Gating strategy was defined using untransfected CHO-S as negative control (blue population). Fluorescence for single clones was tested at generation 20. Q2 gate comprises EGFP+/DsRed+ subpopulation for each clone. **B)** Viable cell density (VCD) and viability of LPs_cMAR (on the left) and LPs_w/o-MAR clones (on the right). Cells were grown in batch culture.


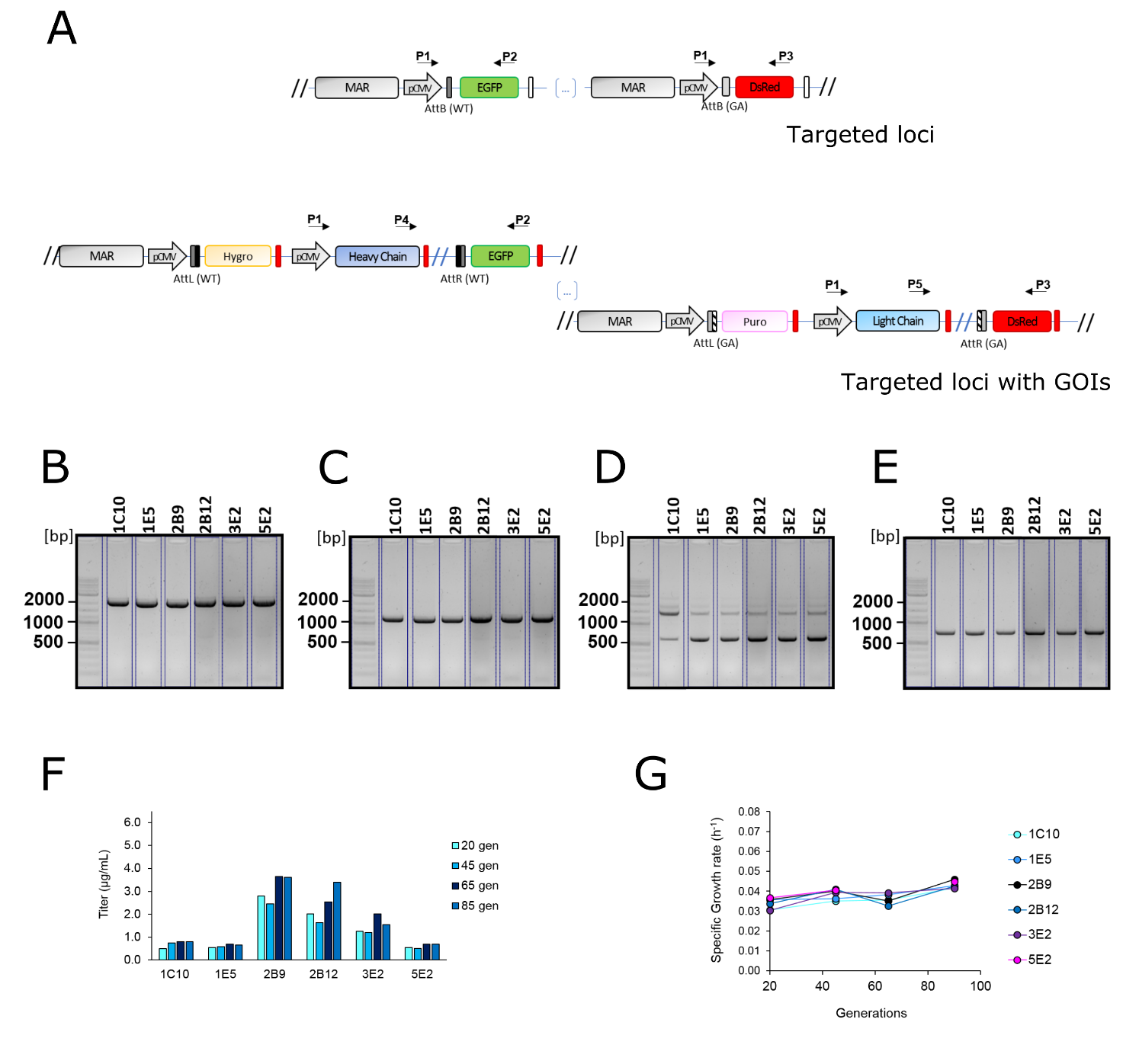
**Figure S2.** Characterization of msAb expressing clones and stability tests. **A)** Set up of genomic PCR for evaluation of SSI on DNA extracted by msAb expressing clones. **B)** Results of genomic PCR using primers P1-P2 (expected amplicon size for integrated HC: 2 kb; expected amplicon size for unoccupied LP_EGFP: 0.5 kb). **C)** Results of genomic PCR using primers P4-P2 (expected amplicon size for integrated HC: 1.2 kb). **D)** Results of genomic PCR using primers P1-P3 (expected amplicon size for integrated LC: 1.5kb; expected amplicon size for unoccupied LP_EGFP: 0.6kb). **E)** Results of genomic PCR using primers P5-P3 (expected amplicon size for integrated LC: 0.8kb). **F-G)** Stability test for mAb expressing clones over 85 generations. Clones were tested for antibody titer and growth. Bars represent antibody titer evaluated on day 7 of fed batch cultures.


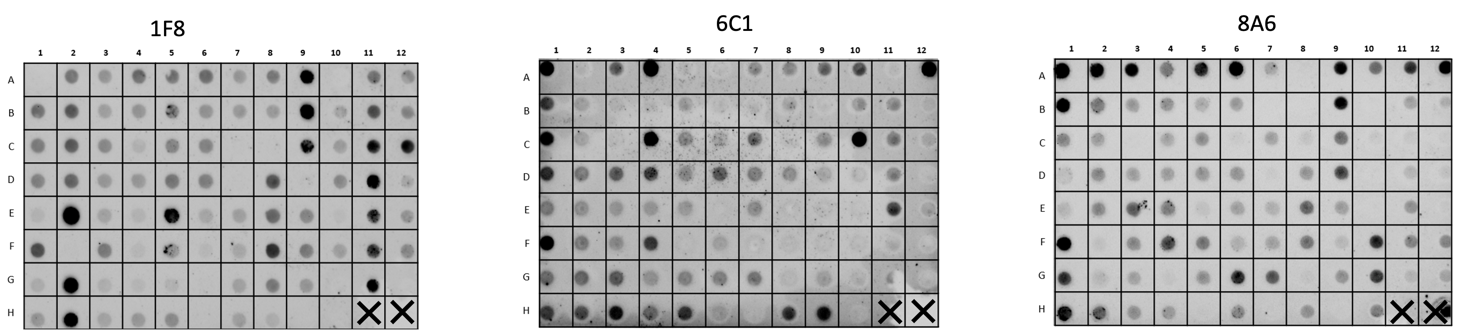


**Figure S3.** Dot-blot analyses for msAb screening using anti-IgG (H+L) detection antibody.


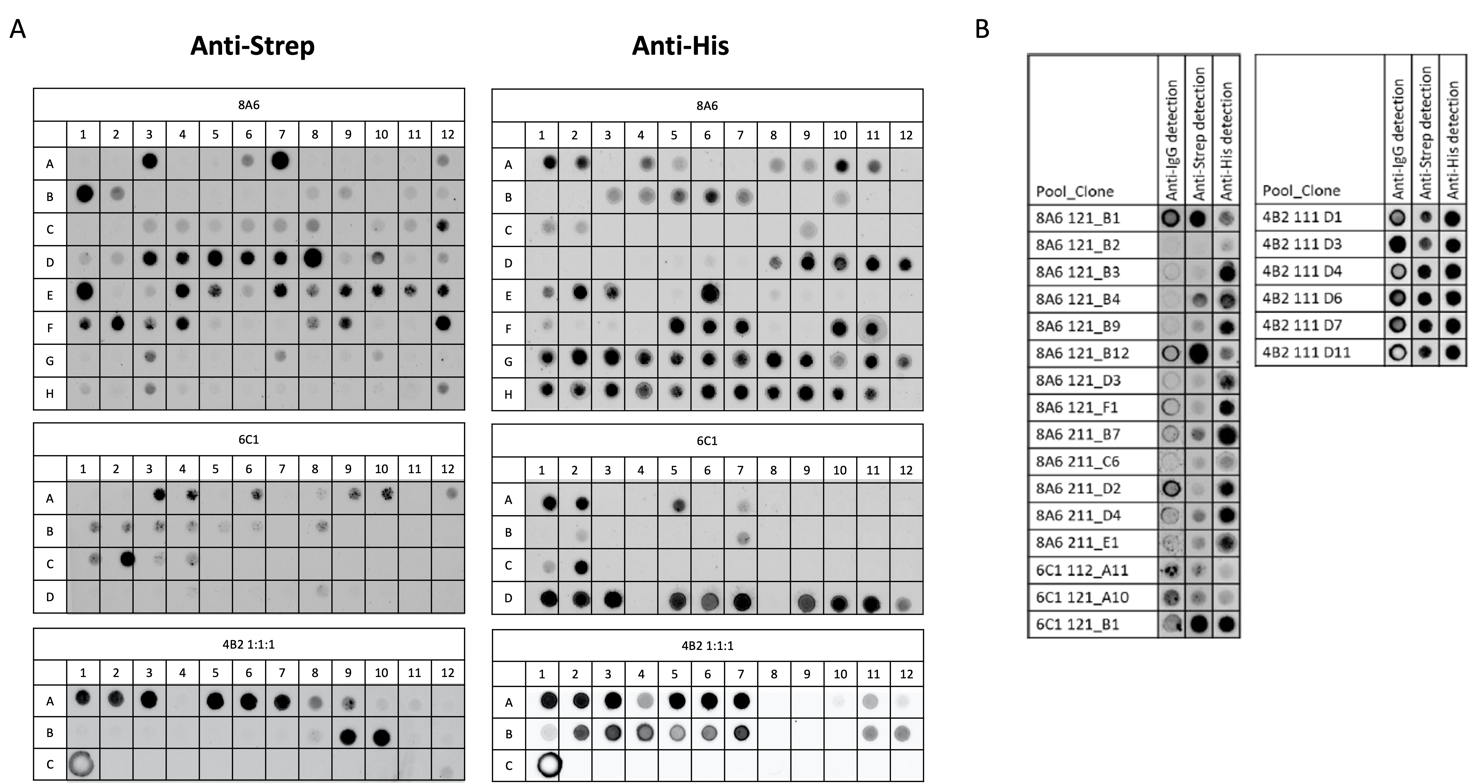
**Figure S4.** Dot-blot analyses for bsAb screening. **A)** dot-blot analyses of picked colonies using Anti-Strep and Anti-His detection antibodies. **B)** dot-blot analyses of productive clones using Anti-Strep, Anti-His, anti IgG (H+L) detection antibodies.

**Table S1.** Data summary for LPs copy number for clones with and without MAR.

| **Clone** | **LP_EGFP copy number** | **LP_DsRed copy number** | **Ratio LP_DsRed/LP_EGFP** |
| --- | --- | --- | --- |
| 1F8 cMAR clone | 5.2 | 36.6 | 7.0 |
| 4B2 cMAR clone | 27.8 | 38.6 | 1.4 |
| 4F9 cMAR clone | 17.2 | 86.5 | 5.0 |
| 6C1 cMAR clone | 9.9 | 54.5 | 5.5 |
| 8A6 cMAR clone | 18.5 | 92.8 | 5 |
| 3A10 w/oMAR clone | 5.3 | 7.8 | 1.5 |
| 3B10 w/oMAR clone | 6.3 | 6.8 | 1.1 |
| 4C9 w/oMAR clone | 5.1 | 10.9 | 2.1 |

**Table S2.** Data summary for GOI copy numbers for msAb expressing clones.

| **Clone** | **HC copy number** | **LC copy number** | **Ratio HC:LC** | **% occupied LP_EGFP** | **% occupied LP_DsRed** |
| --- | --- | --- | --- | --- | --- |
| 1C10 | 4.1 | 4.8 | 0.8 | 78.8% | 13.1% |
| 1E5 | 1.9 | 1.7 | 1.1 | 36.5% | 4.6% |
| 2B9 | 7.8 | 1.9 | 4.1 | 42.2% | 2% |
| 2B12 | 18.2 | 8.9 | 2 | 98.4% | 9.6% |
| 3E2 | 10.6 | 6.9 | 1.5 | 57.3% | 7.4% |
| 5E2 | 3.4 | 6.2 | 0.5 | 34.3% | 11.4% |

**Table S3.** Data summary for GOI copy numbers for bsAb expressing clones. Individual gene copy numbers for knob heavy chain (kHC), hole heavy chain (hHC) and common light chain gene (cLC).

| **Clone** | **kHC copy number** | **hHC copy number** | **cLC copy number** | **% occupied LP_EGFP** | **% occupied LP_DsRed** |
| --- | --- | --- | --- | --- | --- |
| D7 | 20.8 | 7.05 | 26.03 | 100% | 67.4% |
| D11 | 19.05 | 6.64 | 6.84 | 92.4% | 17.7% |

**Table S4.** Summary of fed batch data for msAb expression clones 2B9, 2B12 and 3E2.

| **Clone** | **Medium** | **Max VCD**  **(10^6^ cells/mL)** | **Culture duration (days)** | **Harvest titer (μg/mL)** |
| --- | --- | --- | --- | --- |
| 2B9 | CD CHO 1 | 12.52 | 9 | 4.6 |
|  | CD CHO 2 | 18.75 | 13 | 10.2 |
|  | Balan-CD | 17.81 | 13 | 13.9 |
|  | OptiCHO | 16.62 | 13 | 14.0 |
|  | ActiPro | 20.86 | 10 | 15.8 |
| 2B12 | CD CHO 1 | 14.17 | 9 | 3.4 |
|  | CD CHO 2 | 17.63 | 13 | 6.3 |
|  | Balan-CD | 19.43 | 13 | 14.3 |
|  | OptiCHO | 13.39 | 13 | 11.3 |
|  | ActiPro | 21.81 | 12 | 18.3 |
| 3E2 | CD CHO 1 | 14.38 | 9 | 2.1 |
|  | CD CHO 2 | 14.54 | 12 | 3.0 |
|  | Balan-CD | 20.60 | 13 | 5.5 |
|  | OptiCHO | 12.64 | 13 | 4.6 |
|  | ActiPro | 20.64 | 8 | 8.2 |

**Table S5.** Summary of fed batch data for bsAb expression clones D7 and D11.

| **Clone** | **Medium** | **Max VCD**  **(10^6^ cells/mL)** | **Culture duration (days)** | **Harvest titer (μg/mL)** |
| --- | --- | --- | --- | --- |
| D7 | CD CHO 1 | 6.88 | 10 | 42 |
|  | CD CHO 2 | 14.54 | 14 | 74 |
|  | Balan-CD | 21.48 | 16 | 164 |
|  | OptiCHO | 18.87 | 14 | 196 |
|  | ActiPro | 24.99 | 16 | 300 |
| D11 | CD CHO 1 | 11.24 | 9 | 42 |
|  | CD CHO 2 | 9.23 | 13 | 53 |
|  | Balan-CD | 14.16 | 16 | 147 |
|  | OptiCHO | 12.57 | 12 | 102 |
|  | ActiPro | 16.37 | 14 | 115 |

| **Landing Pad vectors** | | |
| --- | --- | --- |
| **Name** | **Features (5’🡪3’)** | **Comments** |
| pD603 | pCMV- (linear) -BGHpA_pSV40_Neo_SV40pA | ATUM |
| pD603_MCS | pCMV_MCS_BGHpA_ pSV40_Neo_SV40pA | Circularised form of pD603 containing MCS |
| pLP_EGFP | cMAR_pCMV_AttBWT_EGFP_BGHpA | Neomycin resistance gene |
| pLP_DsRed | cMAR_pCMV_AttBGA_DsRed_BGHpA | Neomycin resistance gene |
| pLP_EGFP-w/oMAR | pCMV_AttBWT_EGFP_BGHpA | Neomycin resistance gene |
| pLP_DsRed-w/oMAR | pCMV_AttBGA_DsRed_BGHpA | Neomycin resistance gene |
|  |  |  |
| **Donor vectors** | | |
| pD607 | pCMV- (linear) -BGHpA_pSV40_Hygro_SV40pA | ATUM |
| pD609 | pCMV- (linear) -BGHpA_ pSV40_Puro_SV40pA | ATUM |
| pD607_MCS | pCMV_MCS_BGHpA_ pSV40_Hygro_SV40pA | Circularised form of pD603 containing MCS |
| pD609_MCS | pCMV_MCS_BGHpA_ pSV40_Puro_SV40pA | Circularised form of pD603 containing MCS |
| Donor_Light (mAb) | AttPGA_Puromycin_ SV40pA _pCMV_LC_BGHpA | Promotorless puromycin resistance gene |
| Donor_Heavy (mAb) | AttPWT_Hygromycin_ SV40pA _pCMV_HC_BGHpA | Promotorless hygromycin resistance gene |
| Donor_cLight (bsAb) | AttPGA_Puromycin_ SV40pA _pCMV_cLC_BGHpA | Promotorless puromycin resistance gene |
| Donor_KHeavy (bsAb) | AttPWT_ Hygromycin _ SV40pA _pCMV_kHC_BGHpA | Promotorless hygromycin resistance gene |
| Donor_HHeavy (bsAb) | AttPWT_ Hygromycin _ SV40pA _pCMV_hHC_BGHpA | Promotorless hygromycin resistance gene |
|  |  |  |
| **Other vectors** | | |
| pCAG–NLS–HA– Bxb1 | pCAG_SV40-NLS_HA_BxB1_HGHpA | Addgene #51271 |

**Table S6.** Plasmids used and created in this study

**Table S7** Primers and FISH probes used in this study.

| **gPCR** | |
| --- | --- |
| **Name** | **Sequence (5’🡪3’)** |
| CMV forward (P1) | CAAATGGGCGGTAGGCGTGTACGG |
| EGFP reverse (P2) | TGCGCTCCTGGACGTAGCCTTC |
| DsRed reverse (P3) | TCACGCCGATGAACTTCACCTTGTAGATGAAG |
| Heavy Forward (P4) | CGAGCTGCTTGGCGGCCC |
| Light Forward (P5) | GCAATCCAACAACAAGTATGCTGCCTCC |

| **qPCR - qRT-PCR** | | | |
| --- | --- | --- | --- |
| **Primer set** | **Forward Primer (5’🡪3’)** | **Reverse Primer (5’🡪3’)** | **Probe (5’🡪3’)** |
| B2M | GTG ACA TGG GGC ATG GTG TA | TTG CAC TTG TGG GGG ACC TA | [HEX]CCC CAG CAA GTT GTC ATT TGT CTT TCC CCG T[BHQ-1] |
| eEF1A1 | TCC ACT GGG TCG TTT TGC T | AGC TTT CTG GGC CGA CTT | [HEX]TGC TGG AGC GGG CAA AGT CA[BHQ-1] |
| EGFP | AGC AAA GAC CCC AAC GAG AA | TCG TCC ATG CCG AGA GTG AT | [FAM (Fluorescein)]CCT GCT GGA GTT CGT GAC CGC CGC[BHQ-1] |
| DsRed | AGC TGC CCG GCT ACT ACT AC | GCT CGT ACT GCT CCA CGA TG | [ROX]CCA AGC TGG ACA TCA CCT CCC ACA ACG[BHQ-2] |
| Heavy (mAb) | CCA GCG GCT TTA CTT TCA GC | GGC GTA GTA TGT TGA CCC CC | [FAM (Fluorescein)]GGT CCG CCA GGC ACC CGG CAA[BHQ-1] |
| Light (mAb) | TGA AAG CCG GGG TAG AGA CT | TGG CAG GAG TAT GAC CGA TG | [ROX]GCT GCC TCC AGT TAC CTC AGT CTG ACA CC[BHQ-2] |
| Knob Heavy (bsAb) | CAG AAG TCC CTG TCT CTG | CAA ACT GAG GAT GGC TCC A | [FAM (Fluorescein)]CGG ATC TTG GTC CCA CCC CC[BHQ-1] |
| Hole Heavy (bsAb) | CTT CTT CCT GGT GTC CAA GC | AAT GGT GAT GGT GGT GGT G | [FAM (Fluorescein)] TCC AGA TGG CAG CAG GGC AA[BHQ-1] |
| Common Light (bsAb) | CCT CCA TCT TCC GAG GAA CT | TTG TTG TTG GAC TGC TTG GA | [ROX]GCG CTG TGA CTG TCG CCT GG[BHQ-2] |

| **Generation of FISH probes** | | |
| --- | --- | --- |
| **Primer set** | **Forward Primer (5’🡪3’)** | **Reverse Primer (5’🡪3’)** |
| EGFP | AGA TCC GCC ACA ACA TCG AG | TCG TCC ATG CCG AGA GTG AT |
| DsRed | TCC AAG GTG TAC GTG AAG CA | CTT CTT CTG CAT TAC GGG GC |
| Light Chain | ACA GCA GTA ACC CAG TGG TC | TTC CAG GCT ACG GTA ACA GC |
| Heavy Chain | TGG TAT GTG GAT GGG GTG GA | TGC CCC TTG GCT TTG CTT AT |
